# Data on SARS-CoV-2 events in animals: Mind the gap!

**DOI:** 10.1016/j.onehlt.2023.100653

**Published:** 2023-11-08

**Authors:** Afra Nerpel, Annemarie Käsbohrer, Chris Walzer, Amélie Desvars-Larrive

**Affiliations:** a Unit of Veterinary Public Health and Epidemiology, University of Veterinary Medicine Vienna, Veterinaerplatz 1, 1210 Vienna, Austria; b Wildlife Conservation Society, 2300 Southern Blvd, Bronx, NY 10460, USA; c Research Institute of Wildlife Ecology, University of Veterinary Medicine Vienna, Savoyenstrasse 1, 1160 Vienna, Austria; d Complexity Science Hub Vienna, Josefstaedter Strasse 39, 1080 Vienna, Austria; e VetFarm, University of Veterinary Medicine Vienna, Kremesberg 13, 2563 Pottenstein, Austria

**Keywords:** SARS-CoV-2, Animal hosts, Notification, Data gap, One Health, FAIR data

## Abstract

Current research on SARS-CoV-2 has largely focused on the pandemic's impact on humans, with insufficient attention paid to monitoring, sharing, and communicating information about viral circulation and evolution in animal hosts. The objective of this study was to estimate and characterise the data gap between the number of SARS-CoV-2 cases and related deaths in animals officially notified to the World Organisation for Animal Health (WOAH) via its World Animal Health Information System (WAHIS) and known cases reported through two other data sources: ProMED-mail and scientific papers.

We used the previously published dataset SARS-ANI to retrieve SARS-CoV-2 events in animals published through WAHIS and ProMED-mail. Additionally, we generated SARS-ANI SciLit v1.0, a novel structured dataset of SARS-CoV-2 events in animals published through scientific literature retrieved from PubMed.

We evidenced that at least 52.8% of the SARS-CoV-2 animal cases and 65.8% of the deaths were not reported to WAHIS during 29/02/2020–16/08/2022. Combining information from three different data sources, we compiled a new comprehensive list of 35 animal species reported as susceptible to SARS-CoV-2 under natural conditions, representing a significant advance from the figures reported by the WOAH and the Food and Agriculture Organization of the United Nations. Furthermore, we identified animal species that were underreported to the WAHIS and found that dogs and cats garnered the most attention in research studies. We also showed that, compared to the official WAHIS reports, scientific papers generally experienced longer publication lags and demonstrated that national strategies regarding reporting/publishing of SARS-CoV-2 events in animals greatly differed among countries.

This analysis provides valuable insights into the patterns of reporting animal infections with SARS-CoV-2. The study emphasises the need for improvements in data sharing regarding SARS-CoV-2 events in animals, as this is crucial for effective One Health surveillance, prevention, and control of emerging diseases of zoonotic origin.

## Highlights

• First assessment of missing SARS-CoV-2 animal event data in WAHIS.
• ∼50% of SARS-CoV-2 cases in animals worldwide were not officially reported to WAHIS.
• Combining datasets advances epidemiological knowledge on SARS-CoV-2 in animals.
• Missing data hampers the development of effective One Health interventions.

## 1 Introduction

SARS-CoV-2 is a zoonotic-origin, multi-host pathogen, capable of infecting humans as well as numerous animal hosts [1]. However, to date, research on SARS-CoV-2 has primarily focused on the origin, risk, and impact of the COVID-19 pandemic on humans while neglecting to robustly monitor, share, and communicate SARS-CoV-2 circulation and evolution in a broader One Health context.

In animals, as in humans, infection with SARS-CoV-2 is classified as an emerging disease, and therefore, according to the Terrestrial Animal Health Code [2], all Member States of the World Organisation for Animal Health (WOAH) are required to report cases of animal infection with SARS-CoV-2 to the WOAH. They are also encouraged to share any additional animal health information about this pathogen [2], e.g., from experimental studies or prevalence surveys [3]. Animal cases and related data can be officially notified via the World Animal Health Information System (WAHIS) [4] of the WOAH and publicly shared through the online interface.

Beyond the WAHIS database [4], primary data on SARS-CoV-2 in animals can be retrieved from i) the Program for Monitoring Emerging Diseases (ProMED-mail) [5], which dispatches some information on outbreaks and emerging diseases, selected from email notifications, shared from the WAHIS interface, or gathered from scientific papers and media communications; ii) government websites, which share national data on SARS-CoV-2 cases in animals [6,7]; iii) genomic databases [8,9], which facilitate the sharing and analysis of SARS-CoV-2 genetic data; and iv) preprint and peer-reviewed scientific papers, which typically provide detailed data on experimental studies, field surveys, and case reports. Overall, primary data on SARS-CoV-2 events in animals is dispersed, the data format is heterogeneous, data availability is unsynchronised and interoperability is limited. Furthermore, a single event can be reported in multiple databases, which may lead to inflated or erroneous case and death counts and result in inaccurate estimation of the actual impacts and risks represented by SARS-CoV-2 animal infections.

Real-time data on SARS-CoV-2 in animal and human hosts is critical for assessing pathogen evolution, the threat that novel animal-adapted variants pose to human health, and the risk of spillback events to animal health, conservation, and ecosystem resilience [10]. A significant disparity exists in the availability, quality, and quantity of SARS-CoV-2-related resources and data between humans and animals. This study aimed to quantify and characterise the data gaps between SARS-CoV-2 cases in animals officially notified to the WOAH via WAHIS [4] and known cases reported from two other data sources: ProMED-mail [5] and scientific papers retrieved from PubMed [11].

## 2 Methods

### 2.1 Data on naturally occurring infections with SARS-CoV-2 in animals

Data on natural SARS-CoV-2 infections in animals published through WAHIS [4] and ProMED-mail [5] reports were retrieved from the SARS-ANI dataset [1], which displays weekly updated, structured information on SARS-CoV-2 events in animals. SARS-ANI considered an “event” when *one single case or several epidemiologically related cases were identified by the presence of viral RNA and/or antibodies in an animal* [1]. Therefore, one WAHIS or ProMED-mail report may describe one or more than one SARS-CoV-2 event, and each row of the dataset corresponds to a unique event, which can include one or more than one case. Each event is characterised by a unique identifier and 49 further quantitative and qualitative variables.

To generate a new dataset of SARS-CoV-2 events in animals published in the scientific literature, we conducted a literature search in PubMed [11] using the following query: (SARS-CoV-2[tiab] OR COVID-19[tiab]) AND (animal[tiab] OR animals[tiab] OR zoonoses[tiab]) AND (English[la]) NOT (Review[Publication Type]) NOT (review [tiab]). We included articles describing at least one case of natural SARS-CoV-2 infection in animals that occurred between the beginning of the COVID-19 pandemic (December 2019) and the date of search (24/09/2022). Preprints (since June 2020, PubMed includes preprints resulting from research funded by the National Institutes of Health in its collection [12]) and peer-reviewed papers published in English were included. Reviews were excluded; articles were excluded if they did not deal with SARS-CoV-2, did not evidence SARS-CoV-2 infection (i.e., investigations leading to negative results only), presented results from experimental infections, vaccine or drug tests, or did not directly demonstrate the presence of the virus or antibodies in the animal host (e.g., fur or environmental sampling).

Data collection and dataset structure were calibrated on SARS-ANI [1]. Information was manually extracted from each article, hand-coded, and entered into a dedicated .csv template. Events reported in preprints and subsequently through peer-reviewed papers or published in more than one article were judiciously coded to allow relevant filtering and prevent double counting of the cases or deaths. We additionally extracted information about the date when sampling started and ended. When month and year were given without the day, we assigned the first day of the month for the date when sampling started and the last day of the month for the date when sampling ended. Moreover, since research articles typically involved multiple samples and laboratory tests, we supplemented the base dataset with extra fields dedicated to that type of information.

### 2.2 Data cleaning, quality control, and dataset comparison

Both datasets underwent a data quality control and cleaning procedure as described in Nerpel et al. [1] (Appendix A). Moreover, we manually matched each event published in the scientific literature with its sibling event in SARS-ANI (i.e., a matching event that describes the same SARS-CoV-2 case(s)) by using a pairwise comparison of the events following the method developed for SARS-ANI: firstly, events were filtered by country; we then compared the values populating each field of the new dataset against all values entered in the SARS-ANI dataset for the considered country [1]. The presence of a sibling event in the SARS-ANI dataset was indicated in the new dataset through dedicated fields.

### 2.3 Data analysis

We performed data analysis and generated figures using R v.4.2.3 [13].

We appropriately filtered the SARS-ANI dataset and the newly generated dataset of SARS-CoV-2 events in animals to select subsets of SARS-CoV-2 events that were: i) reported in WAHIS, ii) reported in scientific articles excluding those reported in WAHIS, iii) reported both in scientific articles and in WAHIS, or iv) exclusively reported in ProMED-mail (Appendix B).

Since an event can involve more than one case (or death), we based our comparative analysis on the number of cases (deaths) rather than the number of events. This approach ensured comparability of the data. The total number of cases (deaths) was therefore calculated using three subsets of data that did not overlap: “WAHIS”, “scientific articles excluding WAHIS”, and “ProMED-mail exclusively”. We counted as one individual case (death), each event presenting missing data on the actual number of cases (deaths).

We estimated the number of confirmed susceptible animal species, i.e., species in which the presence of the virus or antibodies against SARS-CoV-2 was evidenced, based on the NCBI-validated [14] scientific names of the hosts, resolved to the lowest taxonomy that could be captured from the information source(s).

Moreover, we calculated the time interval between sampling and publication date for each event published in a research study. The date when sampling ended was used as “sampling date”; when missing, the date when sampling started was used, and the date when the event was confirmed was used if the others were both missing (we preferentially used sampling dates because the date when the case was confirmed was missing in 96.1% of the events). When more than one article described the same event, the earliest date of publication was used for this event.

## 3 Results

### 3.1 Data records

The version of the SARS-ANI dataset [1] we used (GitHub commit 6d03527) included 754 SARS-CoV-2 events in animals recorded from the WAHIS and ProMED-mail databases between 29/02/2020 and 05/04/2023. Dataset fields and possible values are described in Appendix C.

The literature search on PubMed retrieved 3051 peer-reviewed and preprint articles. The newly generated dataset of SARS-CoV-2 events in animals reported in the scientific literature was called “SARS-ANI SciLit” (SARS-CoV-2 events in ANImals retrieved from Scientific Literature). The authors agreed that the first version (v.1.0) of SARS-ANI SciLit would encompass the first 100 eligible hits. Therefore, data from 100 articles, published between 28/03/2020 and 16/08/2022, were included. The structure of the dataset is similar to SARS-ANI [1], where each row represents a SARS-CoV-2 event in animals(s), comprehensively described through 68 attributes (18 more than SARS-ANI) (Appendix C).

Overall, SARS-ANI SciLit v.1.0. displayed 578 SARS-CoV-2 events. Out of the 100 articles, 92 presented original results and were considered for further analyses (eight pairs of papers examined the same events; for each pair, the most updated information was kept).

All events in SARS-ANI and SARS-ANI SciLit v.1.0. comprised at least one missing information point; only 14.6% and 16.4% of the events in the respective dataset had less than five missing information.

### 3.2 Data subsets

Four subsets of data were considered (Appendix B, Table 1). To compare the different subsets of data, only events published from the inception date of each dataset (SARS-ANI: 29/02/2020; SARS-ANI SciLit: 28/03/2020) until 16/08/2022 (maximum date of the smallest dataset, i.e., SARS-ANI SciLit), were considered.

**Table 1:** Description of the data subsets considered in the study. The four subsets cover SARS-CoV-2 events in animals published between 29/02/2020 and 16/08/2022 (study period).

Name of the data subset	Description	No. of events^1^	No. of reports^2^ or papers	No. of unique animal species^4^	No. of taxonomic families	No. of countries	Comments
WAHIS	SARS-CoV-2 events in animals reported to the WOAH and available for consultation via the online public WAHIS platform.	552	259^3^	26	13	33^5^	Events may have also been notified by ProMED-mail and/or described in a scientific paper.
ProMED-mail exclusively	SARS-CoV-2 events in animals reported exclusively by ProMED-mail.	46	29^3^	10	6	16	Events, which, to our knowledge, have not been reported to WAHIS and were not described in scientific papers during the study period.
Scientific papers (all)	SARS-CoV-2 events in animals described in scientific papers (preprints and/or peer-reviewed papers retrieved from PubMed).	501	92^3^	16	6	26	Events may also have been reported to WAHIS and may have been the object of a ProMED-mail report.
Scientific papers excluding WAHIS	SARS-CoV-2 events in animals described in scientific papers but not reported to WAHIS.	333	73^3^	12	6	24	Events retrieved from the scientific literature over the study period, but which, to our knowledge, have not been reported to WAHIS. An event published through a scientific paper may also have been reported through ProMED-mail.

1 We considered an event when one single case or several epidemiologically related cases were identified by the presence of viral RNA (proof of infection) and/or antibodies (proof of exposure) in an animal. Epidemiologically related cases include e.g. animals belonging to the same farm, captive animals housed together, pets belonging to the same household, or animals sampled within the same (generally transversal) study, featuring similar event and patient attributes, i.e. they belong to the same species, underwent the same laboratory test(s) and showed the same results (including variant), exhibited the same symptoms and disease outcome, and were confirmed, reported (when applicable), and published on the same date (e.g. when pets of the same species, sharing the same household, showed different symptoms, they are reported as two distinct events) [1]. In each dataset, each row represents one event which can describe one individual case or several cases that are epidemiologically related.

2 A WAHIS or ProMED-mail report is a document that provides information on animal disease events occurring in a particular country or region.

3 Only unique events were included (those that have been updated by a subsequent event were filtered, see Appendix B).

4 Based on scientific names and resolved to the lowest taxonomic level that could be identified from the information source(s).

5 The last update of the WOAH (9 January 2023) mentioned 36 countries, but we were not able to retrieve this number using the data available from the WAHIS platform because notifications from Belgium, Germany, and Netherlands were done through official mails (called “Situation update”) but, to our knowledge, were not entered on the WAHIS platform. The mails are available at: https://www.woah.org/en/what-we-offer/emergency-preparedness/covid-19/#ui-id-3.

### 3.3 Estimated number of SARS-CoV-2 cases and deaths in animals

We calculated that 1551 cases of infections with SARS-CoV-2 in animals were reported to WAHIS during the study period (29/02/2020–16/08/2022). This is a large underestimate because the number of cases were missing from 73 events (and therefore attributed the value 1), representing 13.2% of the events collected from WAHIS during the study period (53 events involving American mink and 20 involving white-tailed deer). In contrast, 2028 cases were retrieved from scientific papers. The number of individual cases was not reported in 12 events (2.4%), all involving American mink. Of those 2028 cases, 1366 (67.4%) were not reported to WAHIS. Finally, 346 cases of infections with SARS-CoV-2 were reported exclusively through ProMED-mail; the number of individual cases was not reported in 10 events (21.8%), involving American mink (eight events), mule deer, and white-tailed deer (one event each).

**Fig. 1:**
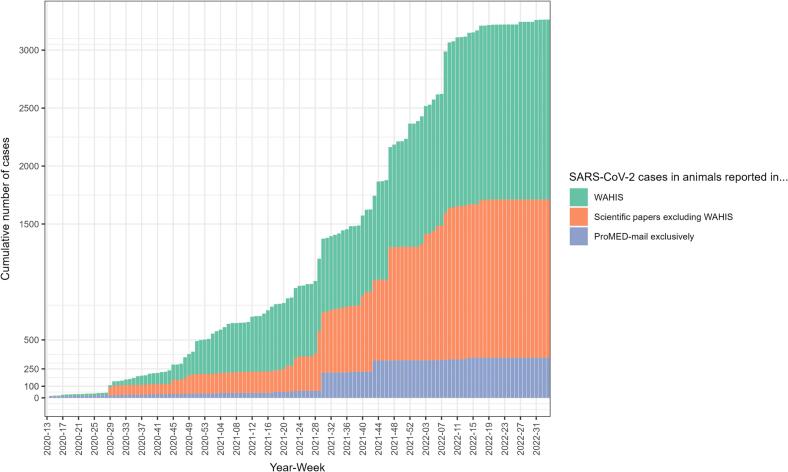
Stacked chart of the estimated cumulated number of SARS-CoV-2 cases in animals, as calculated from data that were reported in WAHIS, in scientific papers excluding WAHIS, and in ProMED-mail exclusively, 29/02/2020–16/08/2022. We counted as one individual case, each event presenting missing data on the actual number of cases.

Overall, we estimated the total number of known SARS-CoV-2 animal cases occurring between 29/02/2020 and 16/08/2022 to be (at least) 3263, with 52.5% not reported to WAHIS (95%CI: [50.8–54.2]) (Fig. 1).

**Fig. 2:**
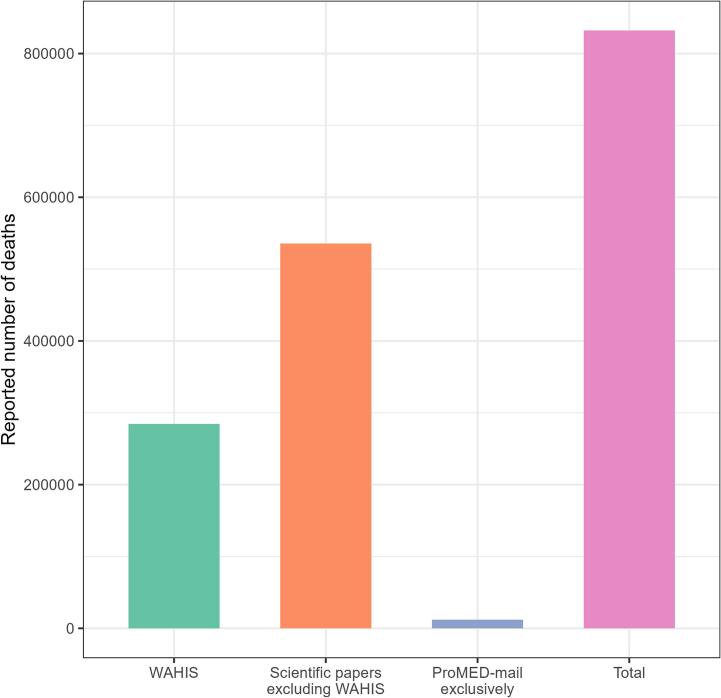
Total number of SARS-CoV-2-related deaths in animals reported through WAHIS, ProMED-mail exclusively, and scientific papers excluding WAHIS, 29/02/2020–16/08/2022. We counted as one individual death, each event presenting missing data on the actual number of deaths.

We estimated the total number of confirmed animal deaths associated with SARS-CoV-2 during the first 2.5 years of the pandemic to be 832,055, of which 65.8% were not reported to WAHIS (95%CI: [65.7–65.9]) (Fig. 2).

### 3.4 Estimated number of animal species susceptible to SARS-CoV-2

We estimated that, during the study period, 35 animal species with resolved taxonomic name (i.e., identified at least at species level), belonging to 16 taxonomic families, were reported as susceptible to SARS-CoV-2 under field conditions (Table 2). This estimate constitutes a considerable evolvement when compared to the 26 species described in the last situation report of the WOAH (31/12/2022) [15]. This estimate also represents a significant advance from the 30 species reported in the last situation update of the Food and Agriculture Organization of the United Nations (FAO, 7/03/2023) [16].

**Table 2:** List of animal species reported as susceptible to SARS-CoV-2 (i.e., by antibodies or virus RNA detection) between 29 February 2020 and 16 August 2022, as extracted from WAHIS reports, ProMED-mail posts, and scientific articles retrieved from PubMed. The table reports the NCBI-resolved scientific and colloquial names, the taxonomic family, the country(−ies) where the species was reported as (sero-)positive, and the reported clinical signs. NS: not specified.

	Scientific name	Colloquial name	Family	Location	Reported clinical signs
1	*Aonyx cinereus*	Asian small-clawed otter	Mustelidae	United States	Respiratory
2	*Arctictis binturong*	Binturong	Viverridae	United States	Subclinical
3	*Canis lupus familiaris*	Dog	Canidae	Argentina; Bosnia and Herzegovina; Brazil; Canada; China; Colombia; Croatia; Denmark; Ecuador; Finland; France; Hong Kong; Italy; Japan; Mexico; Myanmar; Netherlands; Poland; Portugal; Spain; Switzerland; Thailand; Uruguay; United Kingdom; United States	Subclinical; respiratory; nasal discharge; gastrointestinal; neurological; cardiac; weight loss; collapse; myocarditis
4	*Castor fiber*	Eurasian beaver	Castoridae	Mongolia	Respiratory
5	*Crocuta crocuta*	Spotted hyena	Hyaenidae	United States	Respiratory
6	*Equus caballus*	horse	Equidae	United States	Subclinical
7	*Felis catus*	Cat	Felidae	Argentina; Belgium; Bosnia and Herzegovina; Brazil; Canada; Chile; China; Colombia; Croatia; Estonia; Finland; France; Germany; Greece; Hong Kong; Islamic Republic of Iran; Italy; Japan; Latvia; Mexico; Netherlands; Peru; Poland; Portugal; Republic of Korea; Russian Federation; Spain; Switzerland; Thailand; Turkey; United Kingdom; United States; Uruguay	Subclinical; respiratory; cardiac; gastrointestinal; neurological; nasal discharge; tremor; vomiting; sneezing; mild depression; conjunctivitis; ocular discharge; death; myocarditis
8	*Gorilla gorilla*	Gorilla	Hominidae	United States	NS
9	*Gorilla gorilla gorilla*	Gorilla	Hominidae	United States	Subclinical; respiratory; ocular discharge
10	*Hippopotamus amphibius*	Hippopotamus	Hippopotamidae	Belgium	nasal discharge
11	*Lutra lutra*	Eurasian river otter	Mustelidae	Spain	NS
12	*Lynx canadensis*	Canada lynx	Felidae	United States	Respiratory
13	*Lynx lynx*	Eurasian lynx	Felidae	Croatia	Respiratory
14	*Mandrillus sphinx*	Mandrill	Cercopithecidae	United States	Respiratory
15	*Mesocricetus auratus*	Golden hamster	Cricetidae	Hong Kong	Subclinical
16	*Mico melanurus*	Black-tailed marmoset	Cebidae	Brazil	NS
17	*Mustela putorius furo*	Ferret	Mustelidae	Slovenia; Spain; United States	Subclinical; gastrointestinal; respiratory
18	*Myrmecophaga tridactyla*	Giant anteater	Myrmecophagidae	Brazil	NS
19	*Nasua nasua*	Ring-tailed coati	Procyonidae	United States	Subclinical
20	*Neogale vison*	American mink	Mustelidae	Canada; Denmark; France; Greece; Italy; Latvia; Lithuania; Netherlands; Poland; Spain; Sweden; United States	Subclinical; death; respiratory; epistaxis; sudden death; death; gastrointestinal; conjunctivitis
21	*Odocoileus hemionus*	Mule deer	Cervidae	United States	Subclinical
22	*Odocoileus virginianus*	White-tailed deer	Cervidae	United States; Canada	Subclinical
23	*Panthera leo*	Lion	Felidae	Colombia; Croatia; India; Singapore; South Africa; Sweden; United States	Subclinical; respiratory; gastrointestinal; ocular discharge; nasal discharge
24	*Panthera leo bleyenberghi*	Lion	Felidae	Spain	Respiratory
25	*Panthera leo persica*	Lion	Felidae	India	Subclinical; respiratory; nasal discharge; epistaxis
26	*Panthera pardus fusca*	Leopard	Felidae	India	NS
27	*Panthera tigris*	Tiger	Felidae	Argentina; Denmark; India; Sweden; United Kingdom; United States	Subclinical; respiratory; epistaxis; gastrointestinal; ocular discharge; abnormal behaviour; neurological
28	*Panthera tigris altaica*	Tiger	Felidae	United States	Subclinical
29	*Panthera tigris jacksoni*	Tiger	Felidae	United States	Respiratory
30	*Panthera tigris sumatrae*	Tiger	Felidae	Indonesia	Respiratory
31	*Panthera uncia*	Snow leopard	Felidae	United States	Respiratory; gastrointestinal
32	*Prionailurus viverrinus*	Fishing cat	Felidae	United States	Gastrointestinal
33	*Puma concolor*	Puma	Felidae	Argentina; South Africa; United States	Subclinical; respiratory; epistaxis
34	*Saimiri sciureus*	Squirrel monkey	Cebidae	United States	Gastrointestinal; neurological
35	*Trichechus manatus manatus*	Manatee	Trichechidae	Brazil	Subclinical
–	NA	Hamster (unspecified)^1^	Cricetidae	Hong Kong	Subclinical

1 Information provided by the WAHIS reports did not allow to resolve the species taxonomically.

**Fig. 3:**
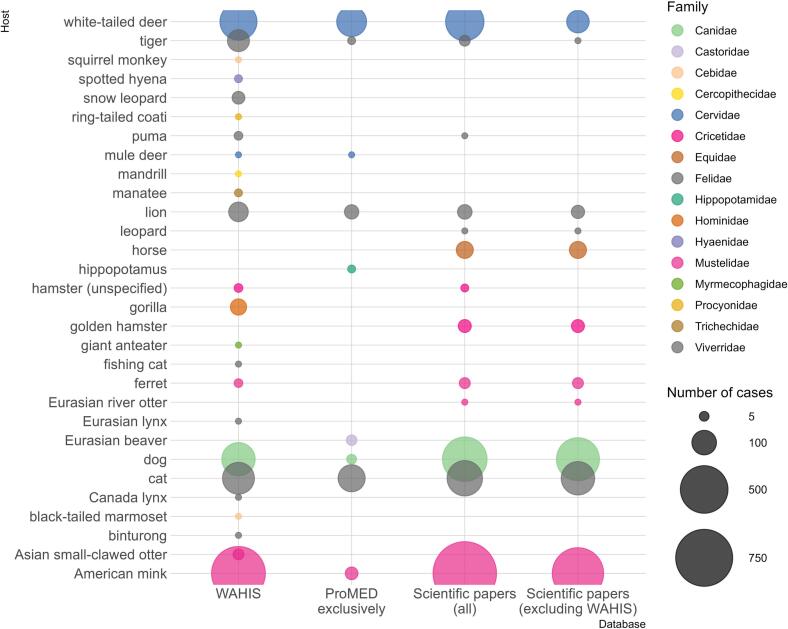
Bubble chart showing the number of cases per animal species (colloquial names are reported on the left side of the figure) reported through WAHIS, ProMED-mail exclusively, scientific papers (all), and scientific papers excluding WAHIS, 29/02/2020–16/08/2022. We counted as one individual case, each event presenting missing data on the actual number of cases.

Among the 35 above-mentioned animal species, six species/subspecies outlined in scientific papers were never recorded on the WAHIS platform (as of date of submission): *Panthera tigris altaica* (Amur tiger)*, Panthera leo bleyenberghi* (Katanga lion)*, Panthera pardus* (leopard)*, Equus caballus* (horse)*, Mesocricetus auratus* (golden hamster)*, and Lutra lutra* (Eurasian river otter). Notably, there was no mention of Equidae in the WAHIS database while the FAO only records experimental infection in horses. Over the study period, only ProMED-mail mentioned SARS-CoV-2 infection in hippopotamus [17] and Eurasian beaver [18]. Although, the hippopotamus cases were reported to WOAH via a letter [19], they could not be retrieved from the WAHIS platform (Fig. 3).

Clinical signs attributed to SARS-CoV-2 in animals were inconsistently reported (Table 2).

### 3.5 Publication lag of SARS-CoV-2 events in scientific articles

On average, over the study period, the time interval between sampling and publication of the SARS-CoV-2 events through scientific papers was 295.7 days (95%CI: [283.9–307.4]). However, we observed a 2.5-fold increase (+254.4%) of this interval between 2020 (139.0 ± 54.5 (SD) days) and 2022 (353.6 ± 146.0). This delay between sampling and dissemination of the results showed little variation in 2020 whereas it was more spread out in 2021 and 2022 (Appendix D). Details about publication lag for the WAHIS platform are reported in Appendix E.

### 3.6 Countries reporting natural infections with SARS-CoV-2 in animals

We identified 44 countries where SARS-CoV-2 events in animals were described (Table 2): Argentina, Belgium, Bosnia and Herzegovina, Brazil, Canada, Chile, China, Colombia, Croatia, Denmark, Ecuador, Estonia, Finland, France, Germany, Greece, Hong Kong, India, Indonesia, Islamic Republic of Iran, Italy, Japan, Latvia, Lithuania, Mexico, Mongolia, Myanmar, Netherlands, Peru, Poland, Portugal, Republic of Korea, Russian Federation, Singapore, Slovenia, South Africa, Spain, Sweden, Switzerland, Thailand, Turkey, United Kingdom, United States, and Uruguay.

This list represents a valuable advance from the WOAH (36 countries) [15] and FAO (40 countries) [16] lists of countries that documented SARS-CoV-2 events in animals. Although covering a longer timeframe (2020−2023), the FAO list does not include Peru, Turkey, China, Iran, Republic of Korea, and Mongolia. However, Egypt and Puerto Rico, both listed by FAO, were not included in our list: the related publication from Egypt is dated 2023 [20] (not covered by our study period) whereas the cases from Puerto Rico (two lions, October 2021 [6]) do not appear on the WAHIS interface.

**Fig. 4:**
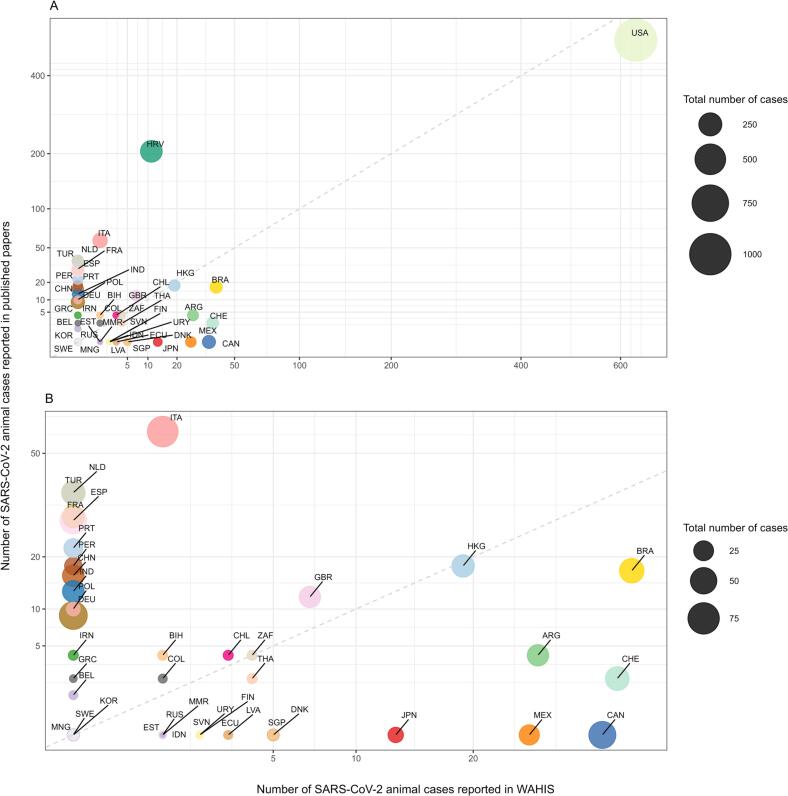
Bubble plots showing, for each country, the number of cases published through scientific papers (all) against the number of cases reported to WAHIS, 29/02/2020–16/08/2022. A: All countries; B: Zoom in on fig. A. To ensure unbiased comparison of the number of SARS-CoV-2 cases reported across countries, the calculation for this figure specifically omitted the cases reported in mink. The size of the dots represents the total number of known cases for each country, estimated by summing up the number of cases reported through WAHIS, ProMED-mail exclusively, and scientific papers (excluding WAHIS). The 45-degree dashed line passing through the origin, with the slope (coefficient) of 1, indicates a one-to-one relationship between the number of cases reported to WAHIS and the number of cases reported in the literature. ARG: Argentina, BEL: Belgium, BIH: Bosnia and Herzegovina, BRA: Brazil, CAN: Canada, CHE: Switzerland, CHL: Chile, CHN: China, COL: Columbia, DEU: Germany, DNK: Denmark, ECU: Ecuador, ESP: Spain, EST: Estonia, FIN: Finland, FRA: France, GBR: United Kingdom, GRC: Greece, HKG: Hong Kong, HRV: Croatia, IDN: Indonesia, IND: India, IRN: Islamic Republic of Iran, ITA: Italy, JPN: Japan, KOR: Korea, LTU: Lithuania, LVA: Latvia, MEX: Mexico, MMR; Myanmar, MNG: Mongolia, NLD: Netherlands, PER: Peru, POL: Poland, PRT: Portugal, RUS: Russia, SGP: Singapore, SVN: Slovenia, SWE: Sweden, THA: Thailand, TUR: Turkey, URY: Uruguay, USA: United States, ZAF: South Africa.

Some countries have disproportionately favoured scientific publications over reporting to WAHIS (e.g., Croatia, Italy, Netherlands, France, Turkey) whereas others have preferentially notified cases to WAHIS (e.g., Brazil, Argentina, Switzerland, Mexico, Japan). The United States, Hong Kong, South Africa, Korea, Sweden, and Mongolia reported the same number of cases in scientific papers and via the WAHIS platform (Fig. 4).

## 4 Discussion

Despite the recommendations of the international institutions [[21], [22], [23]] and obvious benefits (notably for implementing One Health approaches) of collecting and sharing data on SARS-CoV-2 infections in animals, we estimated that more than 50% of SARS-CoV-2 cases and ∼ 65% of SARS-CoV-2 related deaths in animals worldwide were not officially notified to WAHIS. Moreover, data availability on SARS-CoV-2 in animals showed a skew toward Higher-Income economies [24] (we retrieved data from only five countries belonging to Low Income or Lower Middle Income economies [24]).

Events in mink and white-tailed deer were extensively documented over the study period, most likely because the risk of establishment of a reservoir in both species was assessed high, active surveillance at national and regional levels was encouraged [21,[25], [26], [27]] and existing passive surveillance programmes were appropriately used [28]. We identified a reporting gap in the WAHIS platform that mostly affects companion animals, i.e., dogs, cats, and horses, whereas scientific papers presented a substantial bias toward studies involving dogs and cats. Although these animals seem to be dead-end hosts for SARS-CoV-2 [29,30], they raised scientific interest because i) the first SARS-CoV-2 animal case was described in a dog [31]; ii) cat-to-human transmission was evidenced [32]; iii) cats and dogs live closely with humans and their risk of exposure is high; and iv) they are easy to access for research studies.

It is crucial to quantify the gap between known SARS-CoV-2 cases (deaths) and those officially notified to the WOAH because missing data and data imbalance can result in skewed perspectives, e.g., on which species and which geographic areas are mostly affected [30]. Quantifying both the missing data and publication lag has significant impact on epidemic modelling and the evaluation of risk related to animal infections because it can facilitate adjusting for both these limitations by using appropriate methodologies [[33], [34], [35]]. To meet One Health objectives and apply pertinent approaches to address emerging diseases of zoonotic-origin like SARS-CoV-2, we need to improve the quality of reported data (accuracy and validity), reduce reporting lags (timeliness), and invest in specific actions to capture previously unreported cases [36] as well as future ones (completeness). Above all, it is crucial to address the multiple barriers to case notification, in particular, the national *“capacity”* and *“will”* should be explored [37]. It is also urgent to encourage the rapid sharing of scientific results [38] and define novel, adapted communication paths for scientists to report their findings to official institutions (e.g., WAHIS and WHO Hub for Pandemic and Epidemic Intelligence). Publishing and notifying could be paired and complement each other, aligning ethics and scientific recognitions [39].

Lastly, there might also be a need to address the weaknesses in the notification system [40]. Specifically, the inclusion of epidemiological data (e.g., the description of the affected animal population(s), including clinical signs, tests performed, living conditions), which can be highly valuable in evaluating the risks at human-animal-ecosystem interfaces, is discretionary in the Immediate Notifications (INs) form of the WOAH and if provided, such information is typically entered into open-ended fields [3]. Filling in free-text fields during an epidemic might seem overly daunting and time consuming. Proposing closed-ended questions would achieve higher response rates [41], limit the number of possible answers (increasing data consistency), and save time and effort for both the respondent and data analyst [42].

An important limitation of this study lies in the literature search, which was restricted to PubMed. Manually collecting and integrating data in SARS-ANI SciLit necessitates a massive workload. However, searching additional databases and preprint servers would increase the comprehensiveness of the dataset, which, we believe, may be expanded through a collaborative approach and possibly, through integration of AI tools. Nevertheless, validation by a competent (human) operator remains essential. Furthermore, assessing SARS-CoV-2 host range necessitates the compilation, evaluation, and integration of experimental evidence [10], which were not considered in this work. Lastly, we acknowledge that the numbers of cases and deaths are certainly massively underestimated due to undetected cases, unreported/unpublished cases, and a lack of metrics in global farmed mink infections.

## 5 Conclusion

Data (and metadata) sharing following the FAIR principles [43] is one of the key elements of successful One Health surveillance and early-warning programmes. To address zoonotic-origin pandemics and develop robust One Health mathematical modelling and risk assessment frameworks, timely, high quality, and accurate data on both human and animal cases is critical. Such data-driven insights can inform One Health policies and interventions while supporting optimal resource allocation. Anticipating future emerging zoonotic diseases implies establishing robust data streams for near real-time collection and processing of multi-source data. Public health decision and policymaking are increasingly becoming data driven. It is high time to bridge the data gaps.

## Funding

This research did not receive any specific grant from funding agencies in the public, commercial, or not-for-profit sectors.

## CRediT authorship contribution statement

**Afra Nerpel:** Methodology, Investigation, Formal analysis, Data curation, Writing – original draft. **Annemarie Käsbohrer:** Resources, Writing – review & editing. **Chris Walzer:** Conceptualization, Writing – review & editing. **Amélie Desvars-Larrive:** Conceptualization, Methodology, Software, Formal analysis, Data curation, Resources, Writing – original draft, Visualization, Supervision.

## Declaration of Competing Interest

The authors declare no conflict of interest.

## Data Availability

The datasets generated and analysed for this study and the documented R code used to compute results and figures are available on Figshare at: https://doi.org/10.6084/m9.figshare.23264426.v1.

## Appendix Supplementary data

Appendix A Methods for data cleaning and quality control. Image 1

Appendix B Data filtering method. Image 2

Appendix C SARS-ANI and SARS-ANI SciLit field dictionary. Image 3

Appendix D Visualisation of the time interval between sampling and publication of SARS-CoV-2 events in animals through scientific papers. Image 4

Appendix E Time interval between SARS-CoV-2 case confirmation and publication through the WAHIS platform. Image 5
