# Supplementary material for: Data on SARS-CoV-2 events in animals: Mind the gap!
Source: One Health. 2023 Nov 8;17:100653. doi: 10.1016/j.onehlt.2023.100653 (PMC10665207; doi:10.1016/j.onehlt.2023.100653)
Supplement: Appendix A — Methods for data cleaning and quality control. [file mmc1.pdf]

Appendix A. Methods for data cleaning and quality control.

This step was performed in R v.4.2.3 [1].

We checked the value of each field by searching for inaccurate or incorrectly formatted values using the base function *unique()*. Host common and scientific names as well as taxonomy were checked using the functions *gnr_resolve()*, *comm2sci(), sci2comm()*, and *tax_name()* available from the package taxsize [2]. We controlled the presence of duplicates in the newly generated dataset of SARS-CoV-2 events retrieved from published articles (SARS-ANI SciLit) using the following criterion: two (or more) events were considered as duplicates if geolocation (i.e., country, subnational administration, city, location detail), animal host NCBI-resolved common and scientific names, sex, age, symptoms, date when sampling started, date when sampling ended, date of publication, number of cases, number of deaths, the different laboratory tests performed, outcome, ID of the sibling event in the SARS-ANI dataset (when existing), and relationship to another event were identical.

Entries with flagged errors as well as those identified as duplicates were manually inspected against information source(s) and, when necessary, erroneous values were corrected.

References

[1] R: A language and environment for statistical computing (R Foundation for Statistical Computing, Vienna, Austria, 2023). https://www.R-project.org/.

[2] S.A. Chamberlain, E. Szöcs, taxize: taxonomic search and retrieval in R, F1000Res. 2 (2013) 191. https://doi.org/10.12688/f1000research.2-191.v2.
