# Supplementary material for: Data on SARS-CoV-2 events in animals: Mind the gap!
Source: One Health. 2023 Nov 8;17:100653. doi: 10.1016/j.onehlt.2023.100653 (PMC10665207; doi:10.1016/j.onehlt.2023.100653)
Supplement: Appendix B — Data filtering method. [file mmc2.pdf]

Appendix B. Data filtering method.

To avoid double counting of cases and deaths, in both datasets, “updated” events (**related_to_other_entries** = “updated by”) were excluded from the analysis.

SARS-CoV-2 events reported in WAHIS were filtered from the SARS-ANI dataset (**primary_source** OR **secondary_source** = “WAHIS”).

SARS-CoV-2 events reported in ProMED-mail reports exclusively (i.e., not reported by WAHIS and not described in scientific papers) were filtered from the SARS-ANI dataset (**primary_source** = “ProMED” AND **secondary_source** = NA). Moreover, events notified by ProMED-mail which were also described in scientific papers were filtered out, i.e., we excluded an event when the event **ID** in the SARS-ANI dataset was mentioned in the field **SARS_ANI_ID** in the SARS-ANI SciLit dataset.

SARS-CoV-2 events that were described in scientific papers but not reported in WAHIS were filtered from SARS-ANI SciLit dataset by excluding events for which the field **SARS_ANI_ID** contains the **ID** of an event included in the dataset of SARS-CoV-2 events reported in WAHIS (see above). We considered this approach as conservative since it strictly excluded any event that was potentially, fully or partially, reported in WAHIS (events which field **SARS_ANI_comp** = “maybe / more” OR “maybe / different” OR “maybe / less” were excluded). Therefore, this approach tends to underestimate the number of events (and cases) that were published in papers and not reported to WAHIS.

Events published in preprints (**preprint** = “yes”) and subsequently published in peer-reviewed papers were identified and indicated in the field **related_to_other_entries** with the value “updated by” while the corresponding peer-reviewed paper received the value “update of”, allowing appropriate filtering process.

Figure B1. Flow chart showing the data collection process to generate SARS-ANI and SARS-ANI SciLit and further filtering process to obtain comparable datasets for the three databases: WAHIS, ProMED-mail, and PubMed.

See next page.

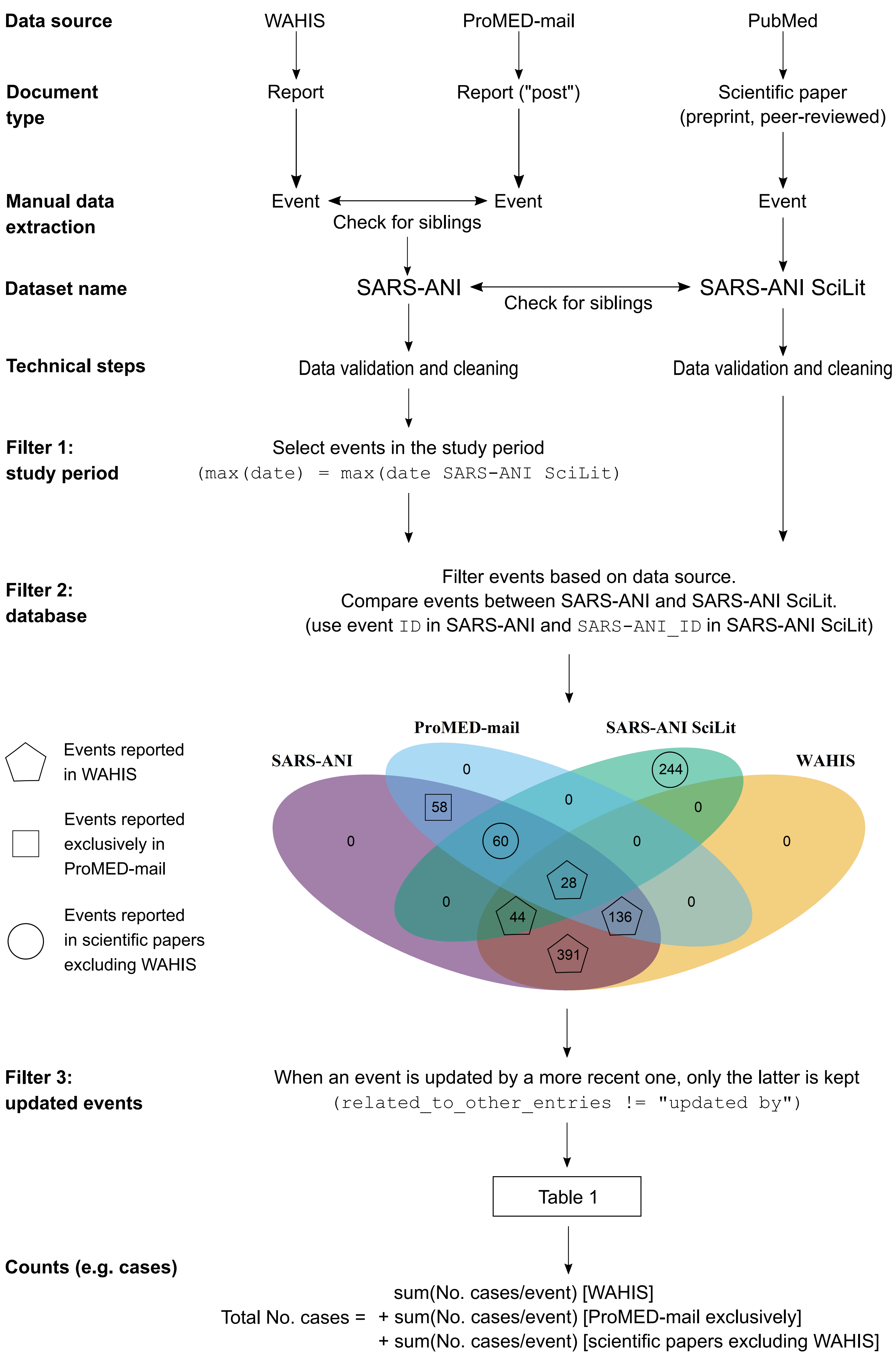

Data source
WAHIS
ProMED-mail
PubMed
Document type
Report
Report ("post")
Scientific paper (preprint, peer-reviewed)
Manual data extraction
Event
Event
Event
Check for siblings
Dataset name
SARS-ANI
SARS-ANI SciLit
Check for siblings
Technical steps
Data validation and cleaning
Data validation and cleaning
Filter 1: study period
Select events in the study period
(max(date) = max(date SARS-ANI SciLit)
Filter 2: database
Filter events based on data source.
Compare events between SARS-ANI and SARS-ANI SciLit.
(use event ID in SARS-ANI and SARS-ANI_ID in SARS-ANI SciLit)
Events reported in WAHIS
Events reported exclusively in ProMED-mail
Events reported in scientific papers excluding WAHIS
SARS-ANI
ProMED-mail
SARS-ANI SciLit
WAHIS
0
244
58
0
0
0
60
0
0
28
0
0
44
136
391
Filter 3: updated events
When an event is updated by a more recent one, only the latter is kept
(related_to_other_entries != "updated by")
Table 1
Counts (e.g. cases)
sum(No. cases/event) [WAHIS]
Total No. cases = + sum(No. cases/event) [ProMED-mail exclusively]
+ sum(No. cases/event) [scientific papers excluding WAHIS]
