# Supplementary material for: Data on SARS-CoV-2 events in animals: Mind the gap!
Source: One Health. 2023 Nov 8;17:100653. doi: 10.1016/j.onehlt.2023.100653 (PMC10665207; doi:10.1016/j.onehlt.2023.100653)
Supplement: Appendix C — SARS-ANI and SARS-ANI SciLit field dictionary. [file mmc3.pdf]

Appendix C. SARS-ANI and SARS-ANI SciLit field dictionary.

Note

We coded with “NS” (not specified) any missing information that should be available but was not specified in the articles (e.g., the scientific name of the animal, the date when infection was confirmed, or outcome of the infection) and with “NA” (not applicable) when the information was not relevant for the event.

To facilitate understanding of the data as well as data integration and comparison, similarly to SARS-ANI [1], we added: the NCBI-resolved [2] common name of the animal host; its NCBI-resolved [2] scientific name (resolved to the lowest taxonomic level, i.e., species or subspecies, depending on the available information); its colloquial name (i.e., the name commonly used to identify the animal in non-specialist language, e.g. “tiger” for “Sumatran tiger” and “Amur tiger”); its scientific name resolved to the species level; and its NCBI-resolved [2] higher taxonomy (i.e., family).

Table C1. SARS-ANI and SARS-ANI SciLit field dictionary. The field description and values are based on those developed for the SARS-ANI dataset [1]. In the table below, **fields** are noted in bold while *field values* are italicized. Asterisks (*) indicate fields that are specific to SARS-ANI SciLit (i.e., not present in SARS-ANI).

Field	Description	Possible string values (when pre-defined) or type of value
**ID**	Unique identifier for each unique event of SARS-CoV-2 in animal(s).	Character string.
**primary_source**	Primary source of information to document the event.	• SARS-ANI: *ProMED*; *WAHIS*.<br>• SARS-ANI SciLit: *PubMed*
**archive_event_number**	Unique identifier for the WAHIS or ProMED-mail report / scientific paper, as provided by the primary source.	• SARS-ANI: WAHIS/ProMED report number.<br>• SARS-ANI SciLit: PubMed Identifier (PMID).
**DOI***	Persistent link to the resource on the Internet.	Digital object identifier (doi) of the scientific paper.
**preprint***	If the paper is a preprint or not (i.e., peer-reviewed).	*yes*; *no*
**link_web**	Link to the online primary source to document the event.	Web resource designed by its Uniform Resource Locator (URL).
**secondary_source**	Secondary source of information to document the event.	• SARS-ANI: *ProMED*; *WAHIS*.<br>• SARS-ANI SciLit: *NA.*
**secondary_source_ID**	Unique identifier for the report, as provided by the secondary source.	• SARS-ANI: WAHIS/ProMED report number.<br>• SARS-ANI SciLit: *NA.*
**secondary_source_web**	Link to the online secondary source for the event.	Web resource designed by its Uniform Resource Locator (URL).
**SARS_ANI_ID***	Event ID as reported in SARS-ANI. Exclusively for events that have a sibling (or matching) event in SARS-ANI.	SARS-ANI ID.

**SARS_ANI_comp***	Specify to which extent the information contained in the scientific article match the information displayed in SARS-ANI.<br>***Note:*** When two events were identified as siblings but there remained uncertainty despite thorough data comparison, events were matched with the mention "maybe" in the relevant field; when information about the compared events were conflicting, the events were not matched.	- *different*: when SARS-ANI and the considered paper describe the same event, but information provided is different and may complete each other;<br>- *less*: when there is less information in the scientific paper than reported in SARS-ANI;<br>- *more*: when there is more information in the scientific paper than reported in SARS-ANI;<br>- *same:* when the information provided in the scientific paper and reported in SARS-ANI is the same (often arises when the scientific paper is a preprint or a peer-reviewed paper that was the object of a ProMED-mail report);<br>- *maybe / more*: when i) retrieved information allow to match the events in a reasonable manner, and ii) the operator, strongly believe, after rigorous event comparison, that the events are siblings, and iii) there is no conflicting information, and iv) there is more information in the scientific paper than in SARS-ANI;<br>- *maybe / different*: when i) retrieved information allow to match the events in a reasonable manner, and ii) the operator, strongly believe, after rigorous event comparison, that the events are siblings, and iii) there is no conflicting information, and iv) information provided is different and may complete each other;<br>- *maybe / less*: when i) retrieved information allow to match the events in a reasonable manner, and ii) the operator, strongly believe, after rigorous event comparison, that the events are siblings, and iii) there is no conflicting information, and iv) here is less information in the scientific paper than reported in SARS-ANI.
**host_com_orig**	Most specific designation of the animal host provided by the source(s), in English.	Character string.
**host_sci_orig**	Scientific name of the animal host as mentioned in the source(s) (scientific names are harmonized so that only the first letter of the genus is capitalized).	Character string.

**host_com_res**	Common name of the animal host, harmonized against the National Center for Biotechnology Information (NCBI) taxonomic backbone.	Character string.
**host_sci_res**	Scientific name of the animal host (resolved to species or subspecies level), harmonized against the National Center for Biotechnology Information (NCBI) taxonomic backbone	Character string.
**host_colloq**	The colloquial name of the host, i.e., the name commonly used to identify the animal in non-specialist language (e.g. “tiger” for “Sumatran tiger”).	Character string.
**host_sci_spec_res**	The scientific name of the host resolved to the species level.	Character string.
**family**	Animal family of the animal host.	Character string.
**epidemiological_unit**	The epidemiological unit considered to describe the event.	- *animal* = one individual;<br>- *group* = a group of animals belonging to the same species housed/living together (excluding farm animals), e.g. zoo animals, pets;<br>- *survey group* = animals belonging to the same species that have been sampled in different locations within the same surveillance programme or survey study;<br>- *farm*: a group of animals belonging to the same species and bred for commercial purposes.
**number_cases**	Reported number of animal(s) tested positive for SARS-CoV-2 in the event.	Integer.
**number_susceptible**	Reported number of susceptible animal(s) of the same species in the event.	Integer.

**number_tested**	Reported number of animal(s) of the same species tested in the event.	Integer.
**number_deaths**	Reported number of direct and indirect death(s) related to the event.	***Note:*** if death is not related to SARS-CoV-2 (see field outcome), number_deaths = 0.
**age**	Age of the animal(s) when tested, in years.	Decimal.
**sex**	Sex of the animal(s).	- *f* = female;<br>- *m* = male.
**country_iso3**	Three-digit ISO country code for the country where the SARS-CoV-2 event was reported.	Character string.
**country_name**	Name of the country where the SARS-CoV-2 event was reported.	Character string.
**subnational_administration**	The subnational administrative region where the SARS-CoV-2 event was reported.	Character string.
**city**	The city where the SARS-CoV-2 event was reported.	Character string.
**location_detail**	Specification of the geographic location enabling to discriminate SARS-CoV-2 events occurring in the same species, at the same date and geolocation (**subnational_administration**, **city**), when the report(s) clearly stipulates that animal(s) were not geolocated at the same place (e.g., different farms or households).	Character string.
**start_date_sampling***	Date when the collect of sample(s) from the animal(s) described in the event began.	Date.<br>***Note:*** When only month and year were provided (i.e., day was missing), we assigned the first day of the month for the date when sampling started.
**end_date_sampling***	Date when the collect of sample(s) from the animal(s) described in the event ended.	Date.

		Note: When only month and year were provided (i.e., day was missing), we assigned the last day of the month for the date when sampling ended, which could then take the value 28, 29, 30, or 31, depending on the year and month considered.
**date_confirmed**	When the SARS-CoV-2 infection or exposure was laboratory confirmed.	Date.
**date_reported**	When the SARS-CoV-2 event was reported by the WAHIS.<br>***Note:*** This information is only provided for event reported through WAHIS reports.	Date (always *NA* in SARS-ANI SciLit).
**date_published**	When the primary source published the SARS-CoV-2 event (**date_published** = **date_reported** when WAHIS is the primary source).	Date.
**related_to_other_entries**	Relationship with another record (see field **related_ID**) in the dataset.	- *new* = the event is not related to any event previously entered in the dataset and no follow-up event exists, but it can be related to an event that was reported on the same day or later in time with one of the following values: **related_to_other_entries** = *living together* or **related_to_other_entries** = *connected* or **related_to_other_entries** = *same study*;<br>- *updated by* = the event has a follow-up event in the dataset, which itself presents the value *update of*. Therefore, a new event gets the value updated by when a follow-up related event is entered;<br>- *update of* = the event is a follow-up of an event previously entered in the dataset;<br>- *living together* = the animal(s) described in the event share(s) the same geolocation (e.g., farm, household, pet store) as another (other) animal(s) that has/have been previously entered in the dataset;<br>- *same study* = the event reports infection in animal(s) belonging to a study that was previously entered in the dataset;

		- *connected* = the event is epidemiologically related to a previously reported event in the dataset (e.g., SARS-CoV-2 events in pet hamsters in pet shops in Hong Kong, following a single importation of infected individuals from the Netherlands);
**related_ID**	Unique identifier (**ID**) of the related entry in the dataset.	Character string.
**test**	First type of laboratory test performed to detect infection with (presence of the virus is evidenced) or exposure to (presence of antibodies is evidenced) SARS-CoV-2.	Character string.
**sampling_type**	Type of sample collected to perform the test (**test**).	Character string.
**test_2**	Second type of laboratory test performed to detect infection with (presence of the virus is evidenced) or exposure to (presence of antibodies is evidenced) SARS-CoV-2.	Character string.
**sampling_type_2**	Type of sample collected to perform the second test (**test_2**).	Character string.
**test_3**	Third type of laboratory test performed to detect infection with (presence of the virus is evidenced) or exposure to (presence of antibodies is evidenced) SARS-CoV-2.	Character string.
**sampling_type_3**	Type of sample collected to perform the third test (**test_3**).	Character string.
**test_4***	Fourth type of laboratory test performed to detect infection with (presence of the virus is evidenced) or exposure to (presence of antibodies is evidenced) SARS-CoV-2.	Character string.

**sampling_type_4***	Type of sample collected to perform the fourth test (**test_4**).	Character string.
**test_5***	Fifth type of laboratory test performed to detect infection with (presence of the virus is evidenced) or exposure to (presence of antibodies is evidenced) SARS-CoV-2.	Character string.
**sampling_type_5***	Type of sample collected to perform the fifth test (**test_5**).	Character string.
**test_6***	Sixth type of laboratory test performed to detect infection with (presence of the virus is evidenced) or exposure to (presence of antibodies is evidenced) SARS-CoV-2.	Character string.
**sampling_type_6***	Type of sample collected to perform the sixth test (**test_6**).	Character string.
**test_7***	Seventh type of laboratory test performed to detect infection with (presence of the virus is evidenced) or exposure to (presence of antibodies is evidenced) SARS-CoV-2.	Character string.
**sampling_type_7***	Type of sample collected to perform the seventh test (**test_7**).	Character string.
**negative_test**	First type of laboratory test mentioned in the report, which outcome was negative.	Character string.
**negative_sampling_type**	Type of sample collected to perform the first test (**negative_test**) that led to negative result.	Character string.
**negative_test_2**	Second type of laboratory test mentioned in the report, which outcome was negative.	Character string.

**negative_sampling_type_2**	Type of sample collected to perform the second test (**negative_test_2**) that led to negative result.	Character string.
**negative_test_3***	Third type of laboratory test mentioned in the report, which outcome was negative.	Character string.
**negative_sampling_type_3***	Type of sample collected to perform the third test (**negative_test_3**) that led to negative result.	Character string.
**negative_test_4***	Fourth type of laboratory test mentioned in the report, which outcome was negative.	Character string.
**negative_sampling_type_4***	Type of sample collected to perform the fourth test (**negative_test_4**) that led to negative result.	Character string.
**reason_for_testing**	Rationale for testing the animal(s).	Character string.
**symptoms**	Reported clinical signs allegedly associated to SARS-CoV-2.	Character string.
**outcome**	Issue of the SARS-CoV-2 infection (or exposure).	Character string.
**living_conditions**	How/where the animal(s) live(s).	Character string.
**source_of_infection**	Most probable source of SARS-CoV-2 infection.	Character string.
**variant**	SARS-CoV-2 genetic variant.	Character string.
**control_measures**	Main intervention(s) implemented to mitigate further spread of the virus.	Character string.
**original_source**	Information source cited by the primary source.	• SARS-ANI: Person, media, or name of the scientific journal reporting the event.

		• SARS-ANI SciLit:: name of the scientific journal in which the paper describing the event was published.
**link_original_source**	Link to the online source cited by the primary source (when applicable).	Web resource designed by its Uniform Resource Locator (URL).

References

[1] A. Nerpel, L. Yang, J. Sorger, A. Käsbohrer, C. Walzer, A. Desvars-Larrive, SARS-ANI: a global open access dataset of reported SARS-CoV-2 events in animals, Sci. Data 9 (2022) 438. https://doi.org/10.1038/s41597-022-01543-8.

[2] C.L. Schoch, S. Ciufo, M. Domrachev, C.L. Hotton, S. Kannan, R. Khovanskaya, et al., NCBI Taxonomy: a comprehensive update on curation, resources and tools, Database 2020 (2020) baaa062. https://doi.org/10.1093/database/baaa062.
