# Supplementary material for: Data on SARS-CoV-2 events in animals: Mind the gap!
Source: One Health. 2023 Nov 8;17:100653. doi: 10.1016/j.onehlt.2023.100653 (PMC10665207; doi:10.1016/j.onehlt.2023.100653)

Appendix D. Visualisation of the time interval between sampling and publication of SARS-CoV-2 events in animals through scientific papers.

Figure D1. Violin plot of the publication lag (i.e., between sampling until publication of the papers) for scientific papers retrieved from PubMed and published in 2020 (n = 12 papers), 2021 (n = 50), and 2022 (n = 25). Study period: 29/02/2020 – 16/08/2022. The vertical blue lines indicate the standard deviation; means are indicated by the blue dots.

In SARS-ANI SciLit v.1.0., the sampling date was missing in 16 papers corresponding to 31 events (i.e., 5.5% of the 564 events considered to estimate the sampling-to-publication time interval).

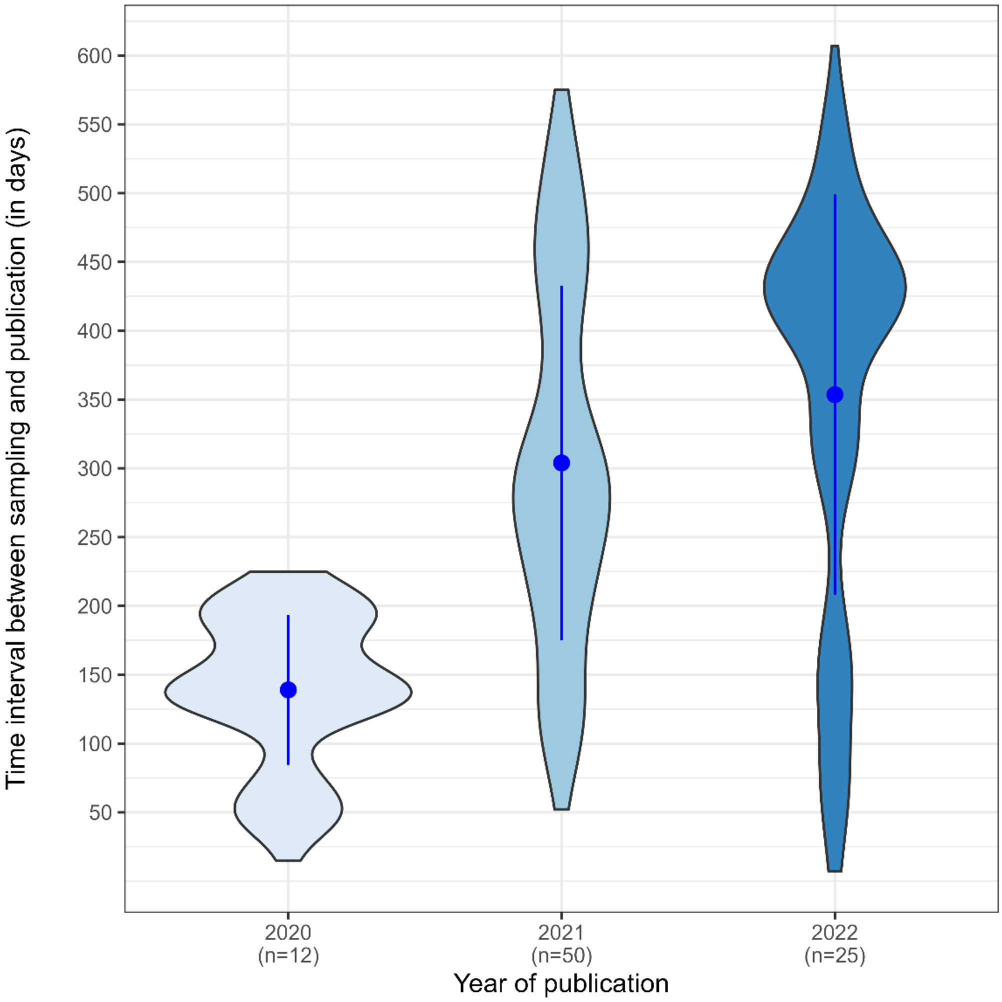

Supplement: Appendix D — Visualisation of the time interval between sampling and publication of SARS-CoV-2 events in animals through scientific papers. [file mmc4.pdf]
