# Supplementary material for: Data on SARS-CoV-2 events in animals: Mind the gap!
Source: One Health. 2023 Nov 8;17:100653. doi: 10.1016/j.onehlt.2023.100653 (PMC10665207; doi:10.1016/j.onehlt.2023.100653)
Supplement: Appendix E — Time interval between SARS-CoV-2 case confirmation and publication through the WAHIS platform. [file mmc5.pdf]

Appendix E. Time interval between SARS-CoV-2 case confirmation and publication through the WAHIS platform.

Methods

Following the methods described in the main article to estimate the publication lag between the date when sampling was conducted and the date when the event was published through a scientific paper, for each event reported in WAHIS, we calculated the time interval between the date when the case was confirmed and the date when the case was published through the WAHIS public interface (the date of sampling could not be retrieved from the WAHIS database).

Results

Over the study period the average time interval between case confirmation and publication through WAHIS was, on average, of 30.1 days (95%CI: [24.6-35.5]) (the date of confirmation was missing in 66 reports corresponding to 241 events, i.e., 43.2% of the 558 events considered to estimate the confirmed-to-publication time interval). However, we observed a near fourfold increase (+384.3%) of this interval between 2020 and 2022 (Table E1).

Table E1. Average time lag between SARS-CoV-2 case confirmation and publication through the WAHIS platform.

Year	Mean $\pm$ SD (standard deviation), in days	Number of reports
2020	15.7 $\pm$ 23.1	41
2021	19.9 $\pm$ 29.0	91
2022	60.2 $\pm$ 112.0	25

Note: While it is not appropriate to directly compare the sampling-to-publication lag (retrieved from published scientific papers) and confirmed-to-publication lag (retrieved from WAHIS reports), we remarked that they differed significantly in terms of their magnitude.
